# Benzo(a)pyrene exposure induced neuronal loss, plaque deposition, and cognitive decline in APP/PS1 mice

**DOI:** 10.1186/s12974-020-01925-y

**Published:** 2020-08-31

**Authors:** Dan Liu, Yujia Zhao, Yuze Qi, Yun Gao, Dezhen Tu, Yinxi Wang, Hui-Ming Gao, Hui Zhou

**Affiliations:** 1 Department of Occupational and Environmental Health Sciences, Peking University, Beijing, 100191 China; 2 Population Health Sciences, German Centre for Neurodegenerative Diseases (DZNE), Bonn, Germany; 3 MOE Key Laboratory of Model Animal for Disease Study, Model Animal Research Center, Institute for Brain Sciences, Nanjing University, 12 Xuefu Road, Nanjing, 210061 Jiangsu Province China

**Keywords:** Benzo(a)pyrene, Alzheimer’s disease, Cognition, Amyloid, Neuroinflammation

## Abstract

**Background:**

Exposure to benzo(a)pyrene (BaP) was associated with cognitive impairments and some Alzheimer’s disease (AD)-like pathological changes. However, it is largely unknown whether BaP exposure participates in the disease progression of AD.

**Objectives:**

To investigate the effect of BaP exposure on AD progression and its underlying mechanisms.

**Methods:**

BaP or vehicle was administered to 4-month-old APPswe/PS1dE9 transgenic (APP/PS1) mice and wildtype (WT) mice for 2 months. Learning and memory ability and exploratory behaviors were evaluated 1 month after the initiation/termination of BaP exposure. AD-like pathological and biochemical alterations were examined 1 month after 2-month BaP exposure. Levels of soluble beta-amyloid (Aβ) oligomers and the number of Aβ plaques in the cortex and the hippocampus were quantified. Gene expression profiling was used to evaluate alternation of genes/pathways associated with AD onset and progression. Immunohistochemistry and Western blot were used to demonstrate neuronal loss and neuroinflammation in the cortex and the hippocampus. Treatment of primary neuron-glia cultures with aged Aβ (a mixture of monomers, oligomers, and fibrils) and/or BaP was used to investigate mechanisms by which BaP enhanced Aβ-induced neurodegeneration.

**Results:**

BaP exposure induced progressive decline in spatial learning/memory and exploratory behaviors in APP/PS1 mice and WT mice, and APP/PS1 mice showed severer behavioral deficits than WT mice. Moreover, BaP exposure promoted neuronal loss, Aβ burden and Aβ plaque formation in APP/PS1 mice, but not in WT mice. Gene expression profiling showed most robust alteration in genes and pathways related to inflammation and immunoregulatory process, Aβ secretion and degradation, and synaptic formation in WT and APP/PS1 mice after BaP exposure. Consistently, the cortex and the hippocampus of WT and APP/PS1 mice displayed activation of microglia and astroglia and upregulation of inducible nitric oxide synthase (iNOS), glial fibrillary acidic protein (GFAP), and NADPH oxidase (three widely used neuroinflammatory markers) after BaP exposure. Furthermore, BaP exposure aggravated neurodegeneration induced by aged Aβ peptide in primary neuron-glia cultures through enhancing NADPH oxidase-derived oxidative stress.

**Conclusion:**

Our study showed that chronic exposure to environmental pollutant BaP induced, accelerated, and exacerbated the progression of AD, in which elevated neuroinflammation and NADPH oxidase-derived oxidative insults were key pathogenic events.

## Background

Alzheimer’s disease (AD) is the most common neurodegenerative disease with progressive loss of memory and other cognitive functions [1, 2]. However, the etiology of AD remains largely unknown. Environmental toxins have been implicated in AD causation [3, 4]. Of special interest, air pollutants have been reported to be associated with acceleration of AD-like pathological changes such as beta-amyloid 42 (Aβ42) accumulation and microglia activation [5].

Benzo(a)pyrene (BaP), the most typical polycyclic aromatic hydrocarbons (PAHs), is a common environmental pollutant derived from incomplete combustion of organic materials, especially petroleum-based fuels and coal; BaP widely exists in ambient air, cigarette smoke, and grilled foods [6, 7]. Because of its high lipophilicity, BaP and its metabolites can across the blood-brain-barrier and deposit in the brain, thus potentially causing neurotoxicity [5, 8]. Emerging evidence has indicated that BaP exposure might be involved in the pathogenesis process of AD. Epidemiological studies have shown that BaP exposure is associated with learning and memory deficits in healthy adults and coke oven workers, which may be due to neurotransmitter alteration [9, 10]. Experimental studies have also shown that BaP exposure in animals induces some AD-like behavior/pathological changes, such as deficits in short-term memory in C57BL/6 J mice [11], accumulation of Aβ42 and neurodegeneration in adult Zebrafish [12], Aβ-related mRNA levels change [13], or tau hyperphosphorylation in SD rats [14].

It is important to investigate whether BaP exposure participates in the disease progression of AD, especially neurodegeneration in the cortex and the hippocampus (two major brain regions affected most in AD), the fundamental feature of AD. In the present study, we provided the experimental evidence that chronic exposure to environmental pollutant BaP induced, accelerated, and exacerbated the progression of AD in both wildtype (WT) mice and APPswe/PSEN1^ΔE9^ transgenic (APP/PS1) mice. Here, APP/PS1 mice, as an early-onset AD mouse model [15], was used to elucidate gene-environment interaction, namely genetic predisposition (mutant APP/PS1 genes) and chronic exposure to environmental pollutant BaP. Primary neuron-glia cultures were treated with BaP and/or aged Aβ peptide (a mixture of monomer, oligomers, and fibrils) to investigate mechanisms of aggravation of Aβ-induced neurodegeneration by BaP exposure.

## Materials and methods

### Animals and treatments

The APPswe/PSEN1^ΔE9^ (also called APP^K670N,M671L^/PSEN1^ΔE9^ or APP^K595N,M596L^/PSEN1^ΔE9^) transgenic (APP/PS1) mice and their wildtype (WT) littermates were obtained from Model Animal Research Center of Nanjing University. All housing and breeding of the animals were performed in strict accordance with the guidelines of the Animal Care and Committee of Nanjing University Animal Center.

**Fig. 1:**
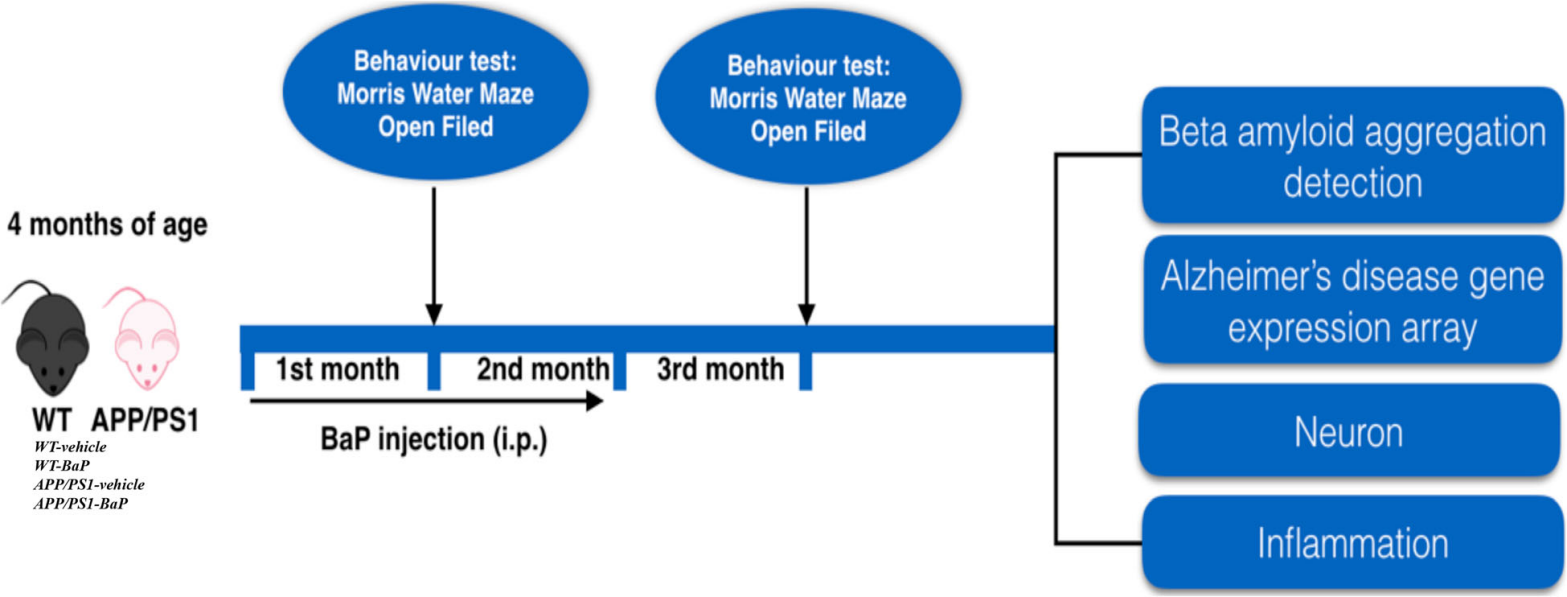
Scheme of experimental design for in vivo studies. BaP (1 mg/kg/bw/day) or corn oil was administered into 4-month-old WT mice and APP/PS1 transgenic mice (WT-vehicle, WT-BaP, APP/PS1-vehicle, APP/PS1-BaP) for 2 consecutive months. Morris water maze and open field behavior tests were performed one month after the initiation/termination of BaP exposure. Then, the mice were sacrificed for gene expression profiling and histological/biochemical analyses 1 month after 2-month BaP exposure

The in vivo experiment was carried out as indicated by the scheme (Fig. 1). Four-month-old APP/PS1 and WT male mice were randomly divided into four groups (WT-vehicle; WT-BaP; APP/PS1-vehicle; APP/PS1-BaP; *n* = 10 each group). BaP was prepared and diluted in corn oil (Sigma, US). In order to study effects of environmental pollutant BaP on AD development, we chose a chronic dosing paradigm (daily intraperitoneal injection for 2 months) and a relatively low dose of BaP (1 mg/kg/bw/day). The dose was chosen based on the environmental/occupational exposure dosage and previous studies [11, 13, 14]. Vehicle groups were received with equal volume of corn oil (50 μl). As for dosing duration and timing of behavior tests and pathological/biochemical examination, we evaluated early onset of cognitive decline of AD after BaP exposure for 1 month (at the age of 5 months) and examined exacerbation of cognitive decline and AD-like pathological/biochemical features of AD at 1 month after BaP exposure for 2 months (at the age of 7 months). Two-month dosing of BaP and 1-month “recovery” time are for better balancing detection of reliable behavior impairment in 7-month-old APP/PS1 mice with possible general toxicity of BaP to the mice and repeated stress from long-time daily dosing procedure before behavior tests. All behavior tests and the result analysis were performed in a double-blinded manner.

### Morris water maze

To investigate spatial learning and working memory abilities, mice were tested in the Morris water maze [16]. The Morris water maze consisted of a large circular plastic pool (diameter 120 cm), which was divided into four quadrants. A circular platform (diameter 10 cm) was located 1 cm below the surface of the opaque water, and the water temperature was kept at 22 °C. Four geometric images, namely a circle, square, triangle, and irregular shape with different colors were stick on the pool wall as visual spatial cues. At the acquisition phase, all mice were trained four trails per day for 5 consecutive days with the submerged platform in a fixed position. For each training trial, the mouse was given 60 s to find the platform. Any mouse that did not reach the platform within 60 s was led to the platform by the experimenter and allowed the mouse to stay on the platform for 10 s. After 24 h from the last training trial, the mice were given a 60-s probe trial without the platform in the pool. Swimming activity of each mouse was tracked via a camera linked to a computer monitoring system. All the parameters were calculated by ANY-maze software.

### Open field test

The open field test measures exploratory behaviors and anxiety-related behaviors. The open field box consisted of four-square blue arenas, 40.5 cm × 40.5 cm × 35.5 cm. The center area was scored as 20 cm × 20 cm area in the center of the open field. The mice were habituated to the behavioral room for 30 min prior to experimental sessions. During the test, the mouse was placed in the center of an arena and left to explore the environment freely for 20 min. Between trials, the arena was cleaned with 70% ethanol and ddH_2_O. All the activities of each mouse were tracked via a camera linked to a computer monitoring system. Average speed, distance traveled, central zone entries, and time spend in the central area were calculated by ANY-maze software.

### Brain tissue preparation

After behavior tests conducted one month after 2-month BaP exposure, the mice were anesthetized using 5% chloral hydrate and perfused transcardially with 0.9% saline; then, brains were harvested. To conduct immunohistochemistry and double-label immunofluorescence, mouse brains were fixed in 4% (w/v) paraformaldehyde (PFA) and then cryopreserved in 30% (w/v) sucrose/phosphate buffer saline (PBS), and stored at 4 °C. The brains were serially sectioned at 10 μm in the coronal plane using Leica VT1200S Fully Automated Vibrating Blade Microtome, and sections were stored in PBS. For biochemical assay and gene array, brain regions were dissected and immediately stored at the − 80 °C. While proteins extracted from the cerebral cortex and the hippocampus were used for Western blot analysis, the total RNA isolated from cortical tissues was used for gene expression profiling.

### Immunohistochemistry, immunocytochemistry, and cell counting

Immunostaining was performed as previously described [17, 18]. Briefly, PFA-fixed cell cultures or brain sections containing the cerebral cortex and the hippocampus were treated with 3% hydrogen peroxide for 10 min followed by three washes with PBS and incubated with blocking solution containing 4% goat serum, 0.4% Triton X-100, and 1% bovine serum albumin (BSA). Brain sections were then incubated overnight at 4 °C with primary antibodies. Human Aβ were detected with 6E10 (1:500, BioLegend, USA, CAT: 803002) antibody. Neurons were stained with an antibody specific for NeuN (neuron-specific nuclear-binding protein; a neuron-specific marker) (1:200, Millipore, USA, CAT: MAB377) or tyrosine hydroxylase (TH, a marker of dopaminergic [DA] neurons) (1:1000, Millipore, MA, CAT: AB152). Microglia and astrocytes were detected with anti-Iba1 (ionized calcium-binding adaptor molecule 1; a microglia marker) (1:500, Wako, Japan, CAT: 019-19741) and anti-GFAP (glial fibrillary acidic protein, as an astroglia marker) (1:800, Millipore, USA, CAT: MAB360) antibody, respectively. Brain sections were incubated with biotinylated anti-rabbit (1:500, Vector Laboratories, USA, CAT: BA-1000) or anti-mouse IgG (1:500, Vector Laboratories, USA, CAT: BA-2000) antibody for 1 h followed by incubation with avidin-biotin complex reagents (Vector Laboratories, USA) for 1 h, and color was developed with 3,3′-diaminobenzidine. Digital images of brain sections were acquired on an Olympus DP72 microscope. Digital images of cultured cells were recorded with a CCD (charge-coupled device) camera.

The optical density of NeuN and GFAP immunoreactivity was measured and analyzed by using Image J software, and a mean value was then deduced by averaging the density of a series of 12 sections that covered the entire cortex/hippocampus. Results were indicated as percentage of WT-vehicle group. The number of Iba-1^+^ cells from a series of 12 brain sections that covered the entire cortex/hippocampus was counted by two investigators individually in a double-blind manner. For visual enumeration of the immunostained cells, images from ten areas per well were randomly taken and the TH-IR neurons were counted. For each experiment, three to four wells per treatment condition were used and results from three to four independent experiments were obtained.

### Thioflavin T staining

To detect Aβ plaques, brain sections containing the cortex and the hippocampus mounted onto glass slides were rinsed in PBS three times and incubated in the humidity chamber for 5 min with the solution of 0.5% thioflavin T (Sigma-Aldrich, USA, CAT: T3516) solution in 0.1 N HCl. Brain sections were then briefly rinsed in PBS and ddH_2_O and cover-slipped with aqueous mounting media. Thioflavin T-stained plaques were viewed with Olympus BX53 semi-motorized fluorescence microscope (excitation wavelength, 488 nm).

### Western blot analysis

The cortex and the hippocampus were gently homogenized in cold RIPA buffer (Radioimmunoprecipitation assay buffer; 50 mM Tris-HCl, pH 8.0, 5 mM EDTA, 150 mM NaCl, 1% Triton X-100, 0.5% sodium deoxycholate, 0.1% SDS, and protease inhibitor cocktail) on ice, centrifuged at 175,000×*g* for 1 h at 4 °C and the soluble supernatant was collected. The protein concentration was determined with Pierce BCA assay kit (ThermoFisher, USA). Equal amounts of total protein (50 μg per lane) were separated on 4~12% Bis-Tris-polyacrylamide electrophoresis gel and transferred to polyvinylidene difluoride membranes. Membranes were blocked with 5% nonfat milk and incubated with the following primary antibodies overnight at 4 °C: anti-NeuN (1:1000, Millipore, USA, CAT: MAB377), anti-Aβ (6E10; 1:1000, BioLegend, USA, CAT: 803002), anti-GFAP (1:1000, Millipore, USA, CAT: MAB360), anti-gp91 (1:1000, BD, USA, CAT: 611414), anti-iNOS (1:1000, Santa Cruz, USA, CAT: sc-7271), and anti-β-actin (1:1000, Cell Signaling Technology, USA, CAT: 8457). The membrane was then incubated with horseradish peroxidase-linked anti-rabbit (1:2000, Cell Signaling Technology, USA, CAT: 7074) or anti-mouse IgG (1:2000, Cell Signaling Technology, USA, CAT: 7076) for 1 h. The blots were detected with enhanced chemiluminescence (ECL) reagent. Images were analyzed by Image J software.

### Alzheimer’s disease gene expression profile array

The gene expression profile related to Alzheimer’s disease was quantified using Alzheimer’s disease gene expression profile array (GeneCopoeia, USA). The gene primer pairs were used for the mRNA expression. In short, the total RNA was isolated from cortical tissues using Trizol reagent following the manufacturer’s direction. An aliquot of RNA (1 μg) was reverse-transcribed into cDNA using the First Strand cDNA synthesis kit (GeneCopoeia, USA). The final cDNA product was used for array using SYBR-Green-based real-time PCR according to the manufactures’ protocol. The amplification was run in the CFX Connect Real-Time PCR Detection system (Bio-Rad, USA). A dissociation curve was used to verify that majority of fluorescence detected was attributed to the labeling of specific PCR products. Relative gene mRNA expression ratios between groups were calculated using the ΔΔCt formulation. All the data were normalized to that of glyceraldehyde-3-phosphate dehydrogenase (GAPDH) in the same sample.

### Primary neuron-glia cultures

Sprague-Dawley (SD) rats were obtained from the Animal Center of Peking University Health Science Center. Mesencephalic primary neuron-glia cultures were prepared from the ventral mesencephalon of embryonic day 14 ± 0.5 SD rats as described before [18]. Briefly, neuron-glia cultures were maintained in MEM (Minimum Essential Medium) supplemented with 10% heat-inactivated fetal bovine serum (FBS) and 10% heat-inactivated horse serum (HS), 1 g/l glucose, 2 mM L-glutamine, 1 mM sodium pyruvate, and 0.1 mM nonessential amino acids. Seven-day-old cultures were treated with vehicle or desirable reagents in the treatment medium (MEM containing 2% FBS, 2% HS, 2 mM L-glutamine and 1 mM sodium pyruvate). At the time of treatment, the neuron-glia cultures were made up of 10% microglia, 50% astrocytes, and 40% neurons of which 3–4% were TH-immunoreactive (IR) neurons.

### Preparation of aged Aβ_1-42_ peptide

Synthesized human Aβ_1-42_ peptide (China peptides, China) was purchased to prepare aged Aβ solution as described before [19]. The Aβ peptide was equilibrated to room temperature for 30 min before dissolved in 100% 1,1,1,3,3,3-hexafluoro-2-propanol (HFIP, Sigma) to 1 mM. After being incubated for 2 h at room temperature, the peptide was dried under vacuum to form peptide films. The peptide film was dissolved in cold sterile phosphate butter saline (PBS) to 100 μM, and after vortexed for 30 s, Aβ solution was incubated at 37 °C for 24 h to form the aged Aβ, which contained Aβ monomer, oligomers, and fibrils.

### Measurement of extracellular superoxide

The production of superoxide was determined by measuring the superoxide dismutase (SOD)-inhibitable reduction of WST-1 (water-soluble tetrazolium 1) as previously described [20]. Neuron-glia cultures grown in 96-well plates were washed and refilled with 150 μl of phenol red-free treatment medium. Fifty microliters of HBSS (Hank's Balanced Salt Solution) containing vehicle or corresponding treatment reagents were then added to each well, followed by addition of 50 μl of WST-1 (1 mM) in HBSS with or without 600 U/ml SOD. The cultures were incubated for 30 min at 37 °C, and the absorbance at 450 nm was read with a SpectraMax Plus microplate spectrophotometer (Molecular Devices, CA).

### Statistical analysis

Data were presented as the mean ± standard error (SE). Differences of parameters in Morris water maze and open filed were detected using repeated measures ANOVA. Statistical significance of other parameters between multiple groups was performed using one- or two-way analysis of variance (ANOVA). When ANOVA showed significant difference, least significant difference (LSD) multiple comparisons post-hoc test was performed. A value of *P* < 0.05 (two-tailed) was considered statistically significant. Statistical analyses were conducted by SPSS 13.0 software (Chicago, IL, USA).

## Results

### BaP exposure induced progressive cognitive decline in WT and APP/PS1 mice

**Fig. 2:**
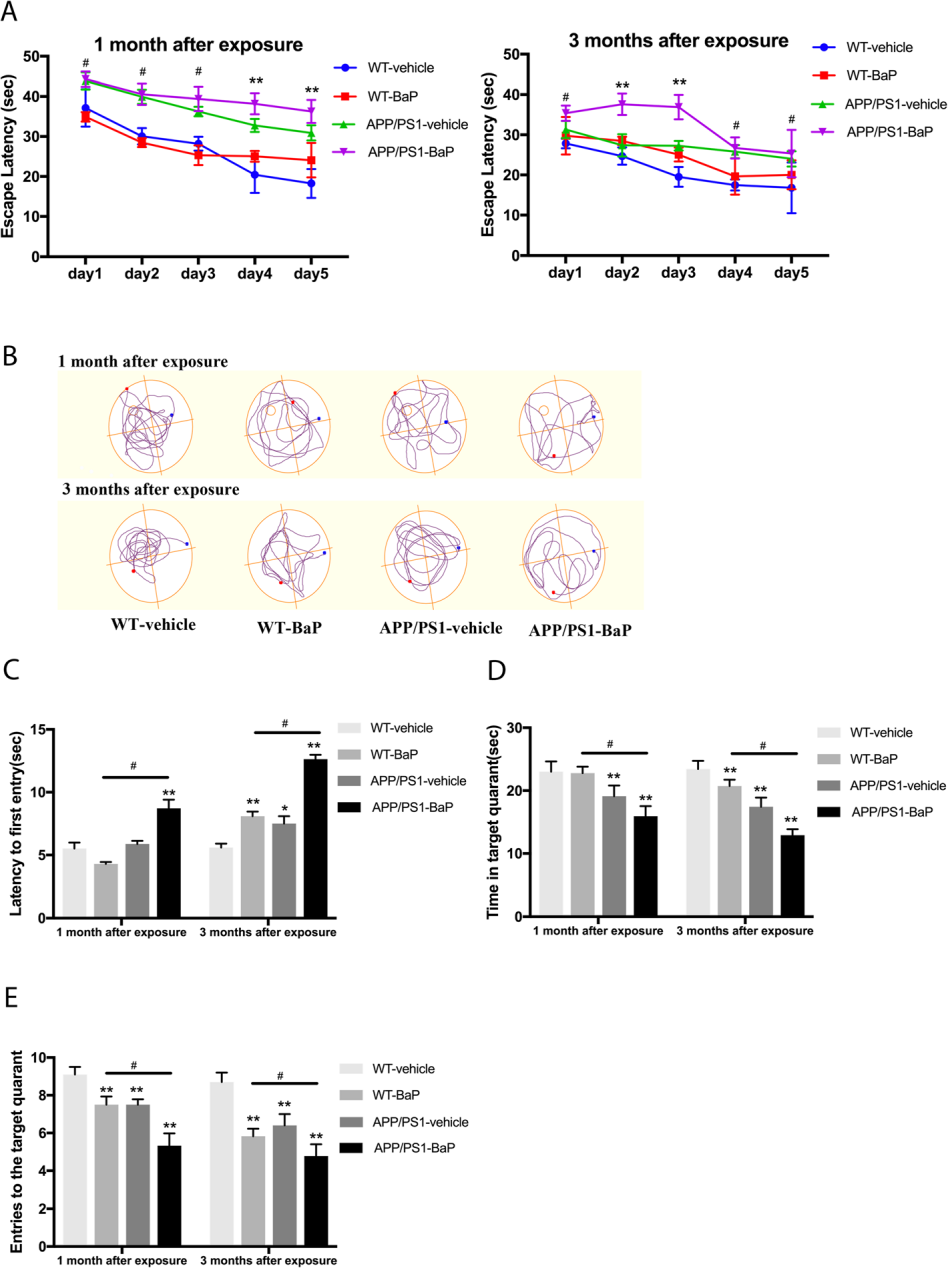
BaP exposure accelerated progressive spatial and working memory decline in the Morris water maze test. **a** Escape latency during 5 days training performed immediately after BaP exposure for 1 month or 1 month after 2-month BaP exposure. **b** Representative path maps of each group in the probe trail. **c** Latency to the first entry to the target quadrant in the probe trail. **d** Time spent in the target quadrant in the probe trail. **e** Entries to the platform quadrant in the probe trail. *N* = 10/group. Data are mean ± SEM, *P < 0.*05* and **P < 0.*01* compared with WT-Vehicle control; ^#^*P < 0*.*05* compared with WT-BaP group

Mouse cognitive functions were evaluated by Morris water maze immediately after BaP exposure for 1 month (at the age of 5 months) and 1 month after 2-month BaP exposure (at the age of 7 months). In the place navigation test, the escape latency to the target platform during the 5-day training period for spatial learning was recorded in Fig. 2a. As an AD animal model, APP/PS1 mice exhibited a longer latency to locate the platform than WT mice. After administration of BaP, the escape latency significantly increased in both WT and APP/PS1 mice (*F = 17*.*80*, *P < 0*.*01*). After training, the hidden platform was removed for the probe test. In the post-training probe test, while the swim paths of WT-Vehicle mice showed directed path-navigation in the target quadrant, the swim paths of WT-BaP mice, APP/PS1-Vehicle mice, and APP/PS1-BaP mice appeared circuitous paths across the area of the pool without obvious preference for the target quadrant (Fig. 2b). After 1-month exposure, APP/PS1-BaP mice showed longer latency to the first entry to the target quadrant (*p* < 0.01) (Fig. 2c), less entry frequency to the target quadrant (*p* < 0.01) (Fig. 2e) and less time in the target quadrant (*p* < 0.01) than APP/PS1-Vehicle mice and WT-BaP mice in the post-training probe test (Fig. 2d). WT-BaP mice showed fewer entries to the target quadrant than WT-Vehicle mice (*p* < 0.01) (Fig. 2e). Two-month BaP exposure made cognitive decline more profound in both WT and APP/PS1 mice (Fig. 2). Thus, BaP induced progressive cognitive deficits in both WT and APP/PS1 mice and caused much severer cognition declines in APP/PS1 mice than WT mice. Moreover, no difference in the swimming speed among all mouse groups (data not shown) indicated that the observed behavior deficits were authentic learning and memory decline but not a result of poor swimming capacity. Collectively, chronic BaP exposure induced, accelerated and/or exacerbated AD-like cognitive decline in WT mice and APP/PS1 mice.

### BaP exposure exacerbated progressive exploratory ability impairment

**Fig. 3:**
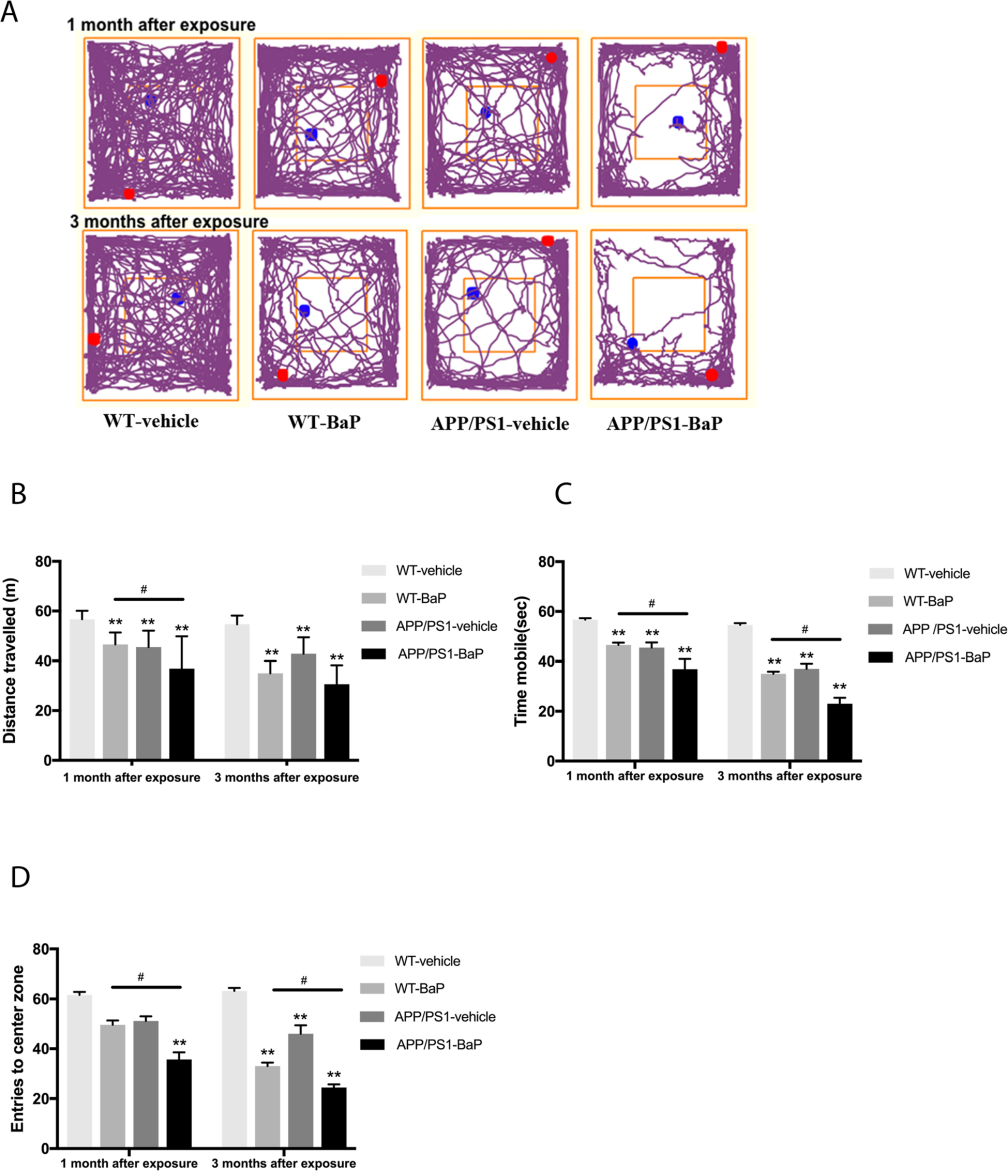
BaP exposure exacerbated progressive exploratory and anxiety impairments. **a** Representative path maps of each group in the open filed test. **b** Total distance traveled during the test. **c** Total time mobile during the test. **d** Entries to the center zone of the open filed. *N* = 10/group. Data are mean ± SEM, **P < 0.*01* compared with WT-Vehicle control; ^#^*P < 0*.*05* compared with WT-BaP group

The exploratory ability and anxiety-related activities were quantified by the open field test. Represented track record images during the test were shown in Fig. 3a. After exposure for 1 month, both WT-BaP and APP/PS1-BaP mice traveled less distance (Fig. 3b), had less mobile time (Fig. 3c) and were less willing to enter the center zone. These behavior impairments further deteriorated after 2-month BaP exposure (Fig. 3d). Altogether, BaP exposure induced progressive exploratory behavior impairment and anxiety-related behavior. Genetic predisposition (mutant APP/PS1 genes) exacerbated such behavior deficits.

### BaP exposure promoted neuronal loss in APP/PS1 mice

**Fig. 4:**
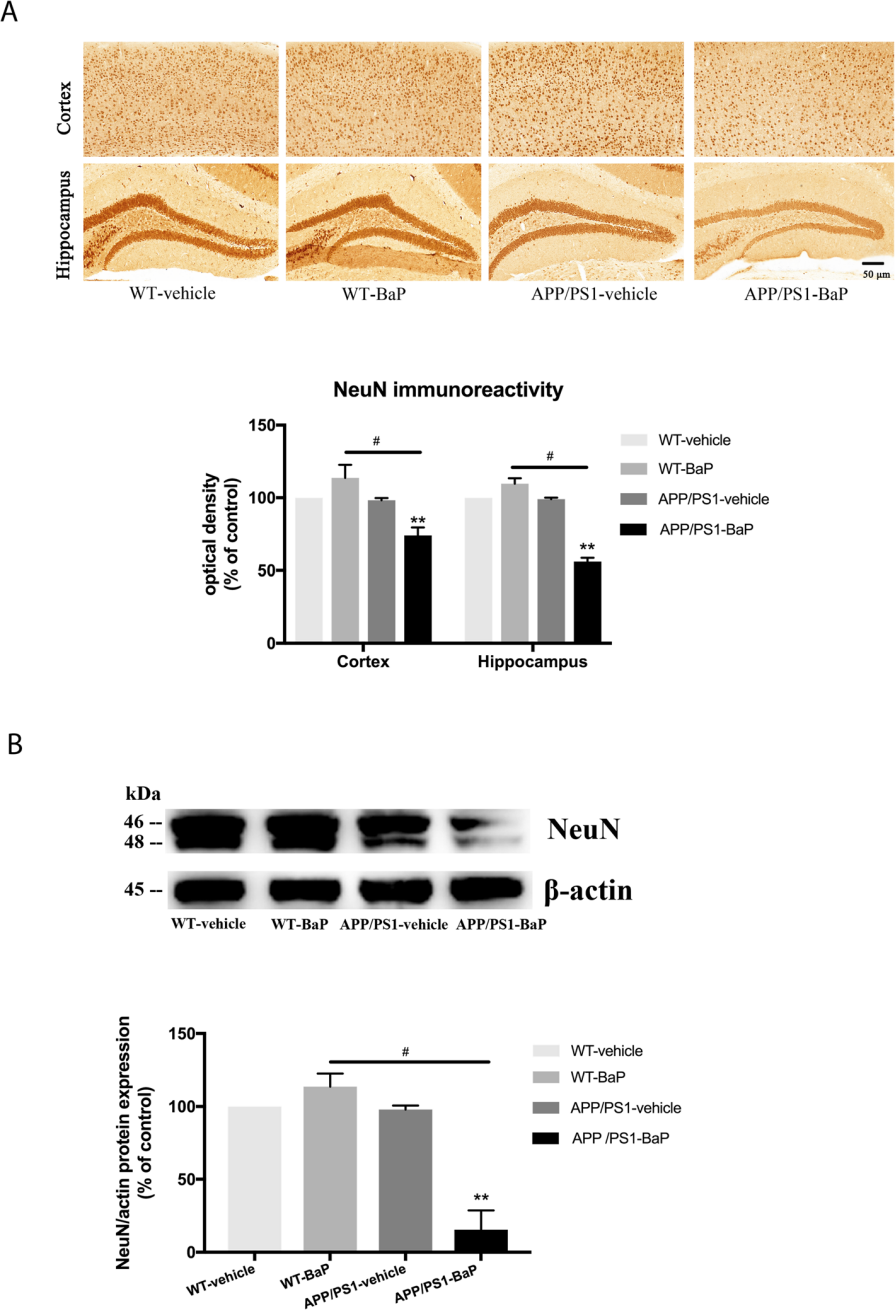
BaP exposure induced neuronal loss only in APP/PS1 mice. **a** Representative images of immunohistochemical staining of NeuN in the cortex and the hippocampus. The quantification results of NeuN immunoreactivity are expressed as a percentage of the corresponding WT-Vehicle control. **b** Levels of NeuN protein in the cortex and the hippocampus were detected by Western blot. β-actin was used to monitor loading errors. Data are expressed as a percentage of the WT-Vehicle control. Data are mean ± SEM of 3–4 mice in each group. Significance was determined by two-way ANOVA followed by LSD multiple comparisons post-hoc test. **P < 0.*01* compared with WT-Vehicle control, ^#^*P < 0*.*05* compared with WT-BaP group

We evaluated the neuronal loss at one month after 2-month BaP exposure in both WT and APP/PS1 mice. As shown in Fig. 4a, BaP induced remarkably neuronal loss by 26% in the cortex (*p* < 0.05) and by 44% in the hippocampus (*p* < 0.05) in APP/PS1 mice. However, no significant neuron loss was shown in WT groups. Significant reduction in the level of NeuN protein in the cortex/hippocampus in APP/PS1 mice after BaP exposure but not in WT groups (Fig. 4b) also supported that genetic predisposition (mutant APP/PS1 genes) and chronic exposure to environmental pollutant BaP together induced synergistic neuronal loss.

### BaP exposure exacerbated Aβ burden and Aβ deposition in APP/PS1 mice

**Fig. 5:**
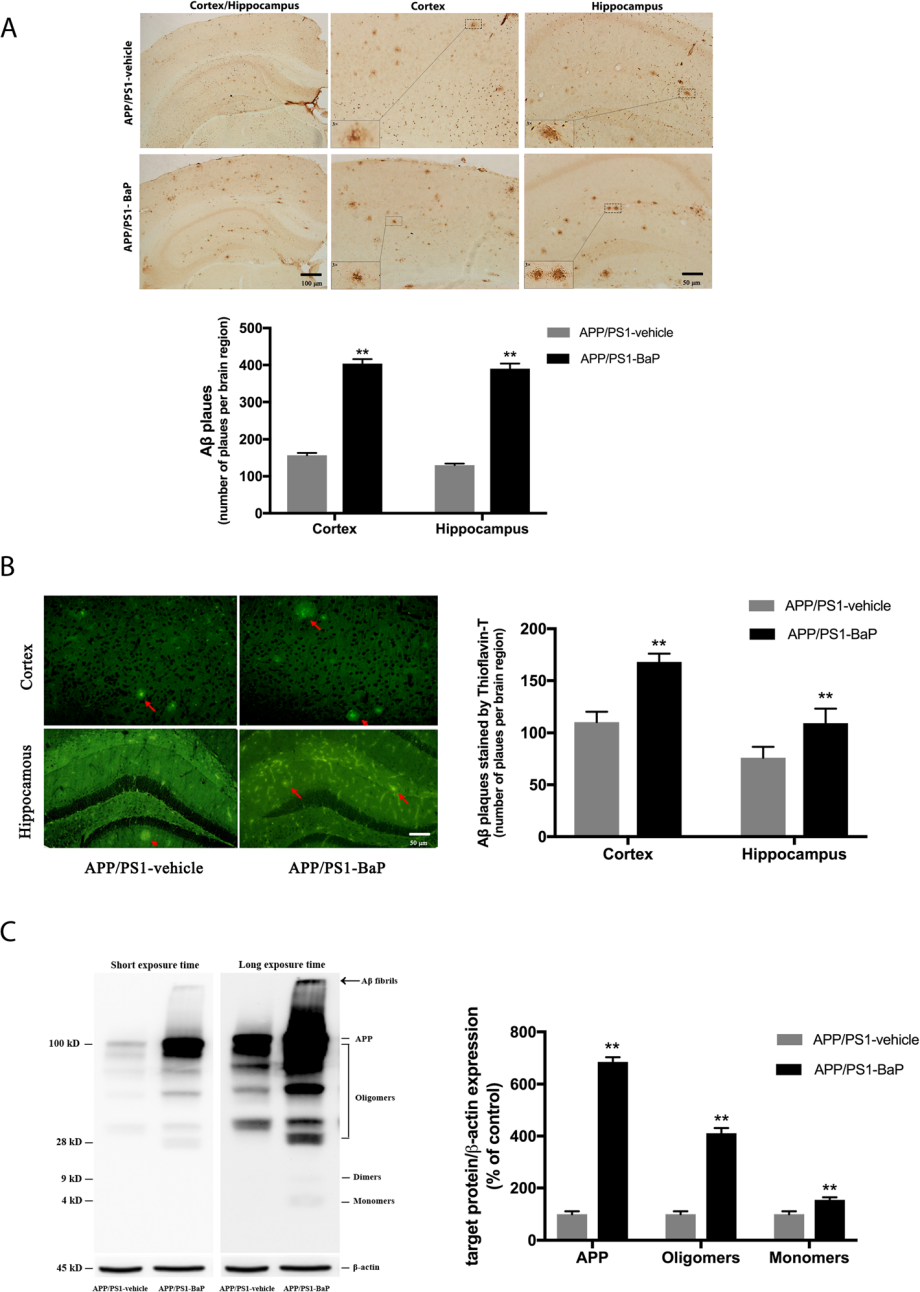
BaP exposure exacerbated Aβ burden and plaque formation in APP/PS1 mice. **a** Representative images of immunohistochemical staining of human Aβ using 6E10 antibody in the cortex and the hippocampus of APP/PS1 mice. Total number of Aβ plaques was counted. **b** Thioflavin-T staining showed Aβ plaques in the cortex and the hippocampus of APP/PS1 mice. Number of Aβ plaques was counted. **c** Levels of RIPA soluble Aβ species and APP were detected by Western blot using 6E10 antibody for human Aβ/APP. β-actin was used to monitor loading errors. The ratio of densitometry values of Aβ monomer (measured in the image with long exposure time), oligomers, and APP (measured in the image with short exposure time) was normalized to β-actin. Data are expressed as a percentage of APP/PS1-Vehicle control and are mean ± SEM of 3–4 mice in each treatment group. Significance was determined by *t* test. **P < 0.*01* compared with APP/PS1-Vehicle. Arrow indicates Aβ fibrils stuck in the loading well of the SDS-PAGE gel and then transferred to the PVDF membrane

Since APP/PS1 mice were considered as the ideal model for analyzing amyloidosis in the pathogenesis of AD, effects of BaP exposure on amyloidosis were elucidated by immunohistochemistry, Thioflavin-T (ThioT) stain, and Western blot assays. Immunohistochemistry analysis of Aβ deposition showed that BaP exposure significantly increased plaque burden by more than 2-fold in both cortex and hippocampus (*p* < 0.05) in APP/PS1 mice (Fig. 5a). The ThioT-positive Aβ compact plaques increased by 53% in the cortex (*p* < 0.05) and by 44% in the hippocampus (*p* < 0.05) (Fig. 5b). These findings indicated that BaP exposure exacerbated a key pathological feature of AD, formation of Aβ plaques and Aβ fibrils. Because more and more studies have documented a pivotal role of Aβ oligomers in the pathogenesis of AD, we used the RIPA buffer to extract soluble Aβ monomers and oligomers. Western blot results showed a dramatic increase in Aβ oligomers in the cortex/hippocampus of APP/PS1 mice after BaP exposure (Fig. 5c). The level of Aβ monomer and amyloid precursor protein (APP, a 770 amino acid protein with a molecular mass of ~ 100 kDa, which can be detected by 6E10 antibody) also significantly increased (Fig. 5c). Collectively, these results indicated BaP exposure exacerbated accumulation of both soluble Aβ monomer and oligomers and insoluble Aβ fibrils in the cortex and the hippocampus of APP/PS1 mice.

### BaP exposure altered AD-related gene expression profiles

In this study, the mouse Alzheimer’s Disease RT^2^ Profiler PCR Array was used to analyze the BaP-induced alternations of gene expression profiles in the cortex of both WT and APP/PS1 mice. Among 101 tested genes (Supplemental table 1), the ones with differential expression in WT-BaP, APP/PS1-Vehicle, and APP/PS1-BaP mice compared with WT-Vehicle (Table 1) were linked to Aβ generation, clearance and degradation, neuroinflammation, and neuronal damages. In general, the BaP-induced alternation of gene expression profiles was more profound in APP/PS1 mice than that in WT mice. Although no clear amyloidosis was observed in WT-BaP mice (data not shown), gene expression profiles showed significant changes in multiple genes related to Aβ generation, clearance, and degradation in WT mice upon BaP exposure. Notably, gene expression changes and resultant functional alterations of the corresponding proteins in WT-BaP mice could be partial reason for neurobehavioral deficits in WT-BaP mice and could represent the initial phase of the incipient AD. Genetic predisposition (mutant APP/PS1 genes) facilitated the BaP-induced pathogenic progress.

**Table 1:** The differential Alzheimer’s disease gene expression profiles in WT and APP/PS1 mice after BaP exposure

Category	Gene	WT-vehicle (fold change)	WT-BaP (fold change)	APP/PS1-vehicle (fold change)	APP/PS1-BaP (fold change)
**Inflammatory and immunoregulatory process**	Interleukin 1 alpha (Il1a)	1.00	0.97	0.90	1.92^*#^
	Interleukin 1 beta (IL1β)	1.00	0.65^**^	4.01^**^	4.67^**#^
	Interleukin 3 (IL3)	1.00	3.75^*^	2.82^*^	3.81^*^
	Interleukin 6 (Il6)	1.00	0.94	1.30	2.14^*#^
	gp91	1.00	3.19^*^	4.88^*^	5.71^*^
	p67	1.00	1.51	2.75^*^	2.76^*^
	p47	1.00	0.99	1.42	1.36
	p40	1.00	1.17	1.97^*^	1.77^*^
	iNOS	1.00	0.58^*^	0.75	1.41^*#^
	CD36 antigen (Cd36)	1.00	2.65^**^	2.58^**^	7.41^**#^
	Chemokine ligand 5 (Ccl5)	1.00	1.03	4.19^*^	2.91^**#^
	Chemokine ligand 7 (Ccl7)	1.00	1.25	2.14^*^	1.56^*^
	Tumor necrosis factor (Tnf)	1.00	1.70^*^	2.61^**^	10.19^**#^
	Arginase (Arg1)	1.00	0.94	1.40	5.61^*#^
	TREM2	1.00	1.06	1.67^*^	5.86^*#^
	Prostaglandin-endoperoxide synthase 2 (Ptgs2)	1.00	1.60^*^	0.92	2.89^*^
**Secretases**	Amyloid beta precursor protein (APP)	1.00	2.51^*^	6.56^*^	8.15^*#^
	Beta-site APP cleaving enzyme 1 (Bace1)	1.00	1.24^*^	2.10^*^	2.93^*#^
	Presenilin 1 (Psen1)	1.00	0.97	2.07^*^	2.93^**#^
	Presenilin 2 (Psen2)	1.00	0.93	1.27	1.88^*#^
	Nicastrin (Ncstn)	1.00	1.04	2.61^**^	3.24^**#^
	Anterior pharynx defective 1b homolog (Aph1b)	1.00	1.44^*^	2.78^**^	3.20^*#^
	Presenilin enhancer 2 (Pen2)	1.00	1.15	1.11	1.15
**Beta-amyloid degradation**	Membrane metallo endopeptidase	1.00	1.77^*^	1.32	4.75^*#^
	Plasminogen activator (Plau)	1.00	1.05	3.13^*^	3.97^*#^
	Insulin degrading enzyme (IDE)	1.00	1.04	2.53^**^	3.16^*#^
	Neprilysin (NEP)	1.00	2.53^*^	0.29^*^	0.52^*#^
**Synaptic formation**	Zinc transporter (Slc30a3)	1.00	1.75^**^	0.86	2.81^*^
	Aplipoprotein E (Apoe)	1.00	2.17^*^	0.85^*^	1.49^*^
	Brain-derived neurotrophic factor (Bdnf)	1.00	1.43^**^	3.34^**^	3.65^**#^
	Nerve growth factor receptor (Ngfr)	1.00	1.59^*^	2.64^*^	5.49^**#^
	Neurotransmitter transporter (Slc6a4)	1.00	0.84	0.25^*^	0.48^**^
	Agrin (Agrn)	1.00	1.32	1.39	0.48^*#^
	Glial cell line-derived neurotrophic factor (Gdnf)	1.00	1.26	1.32^*^	2.74^*^
	Acetylcholinesterase (Ache)	1.00	1.33^*^	9.31^*^	5.14^*#^

Differential expression values for a subset of genes, grouped by functional categories, for WT and APP/PS1 mice. Values were measured by array analysis and were expressed as fold changes compared with control group. Fold changes were in linear scale, thus < 1.0 means downregulated genes while > 1.0 means upregulated. *P < 0.*05*, ** P < 0.*01* compared with control, ^**#**^*P < 0*.*05*, compared with WT-BaP mice

Pathways and functional systems of the differentially modulated genes were further analyzed. Among the highlighted categories, inflammation and immunoregulatory process, Aβ secretes, and degradation and synaptic formation pathways/systems emerged as the most robustly altered in AD progression after BaP exposure. As shown in Table 1, almost all the Aβ secretion-related proteins tested in this study were significantly increased in APP/PS1 mice after BaP exposure but not in WT mice. For instance, in APP/PS1 mice, beta-secretase 1 (Bace1, as known as beta-site APP cleaving enzyme 1), presenilin proteins (Psen1/2), and aph-1 homolog b (Aph1b) were raised to 2.93-, 1.88-, and 3.20-folds respectively after BaP exposure. However, mild changes of these proteins in WT-BaP mice (Table 1) suggested that BaP facilitated abnormal Aβ secretion in APP/PS1 mice, which was in concordance with accelerated/exacerbated Aβ burden observed in APP/PS1-BaP mice (Fig. 5c). Interestingly, Aβ degradation-related proteins such as membrane metalloendopeptidase (MME), plasminogen activator (Plau), and insulin degrading enzyme (IDE) were also elevated in APP/PS1-BaP mice, but not in WT-BaP mice, which is consistent with deteriorated Aβ burden in APP/PS1 mice after BaP exposure.

The gene expression profiles related to synaptic formation were also remarkably changed in BaP-treated mice. Agrin, known to be important for synaptic differentiation, formation, and/or maintenance [21–23], was significant decreased in APP/PS1 mice after BaP exposure (Table 1). Altered expression and abnormal distribution of agrin in AD brain contribute to AD pathogenesis [21]. Slc6a4, a gene encoding SERT (serotonin transporter), was significantly downregulated in APP/PS1 mice with or without BaP exposure (Table 1). The brain of AD patients show reduced SERT in both protein and mRNA, and reduced SERT is associated with depression and anxiety in AD [24, 25]. Acetylcholinesterase (AChE) was remarkably upregulated in WT and APP/PS1 mice after BaP exposure (Table 1). Studies have shown increased AChE activity in plasma and elevated AChE levels around amyloid plaques in AD patients [26, 27]. Interestingly, neurotropic factors BDNF (brain-derived neurotrophic factor) and GNDF (glial cell line-derived neurotrophic factor) were upregulated in WT and APP/PS1 mice, which could be protective responses to BaP-induced neuronal damages. These findings together suggested that BaP exposure might induce synaptic loss/dysfunction.

It is notable that neuroinflammatory responses were the pathway with the most robust alteration after BaP exposure in both WT and APP/PS1 mice. Neuroinflammatory cytokines (e.g., TNFα, IL3, TREM2, IL1β, and IL6) were slightly increased in WT-BaP mice, but remarkably upregulated in APP/PS1-BaP mice, which indicated persistent neuroinflammation in BaP-induced APP/PS1 mice. In addition, multiple subunits of NADPH oxidase including gp91, p67, p40, and p47 were remarkably upregulated. In particular, gp91 (the catalytic subunit of NADPH oxidase) was upregulated by 3.19-folds and 5.71-folds in WT-BaP mice and APP/PS1-BaP mice, respectively (Table 1). Taken together, these alternation patterns of gene expression in WT and APP/PS1 mice after BaP exposure indicated that BaP could initiate AD-related pathological and biochemical pathways to precipitate in AD progression. Notably, sustained neuroinflammatory responses may play a crucial role in BaP-/Aβ-induced AD-like phenotypes. Neuroinflammation has been believed to be the key molecular event in the initial phase of AD; We next investigated how neuroinflammation and NADPH oxidase activation affected BaP’s aggravation of Aβ-induced AD progression.

### BaP exposure induced neuroinflammation

**Fig. 6:**
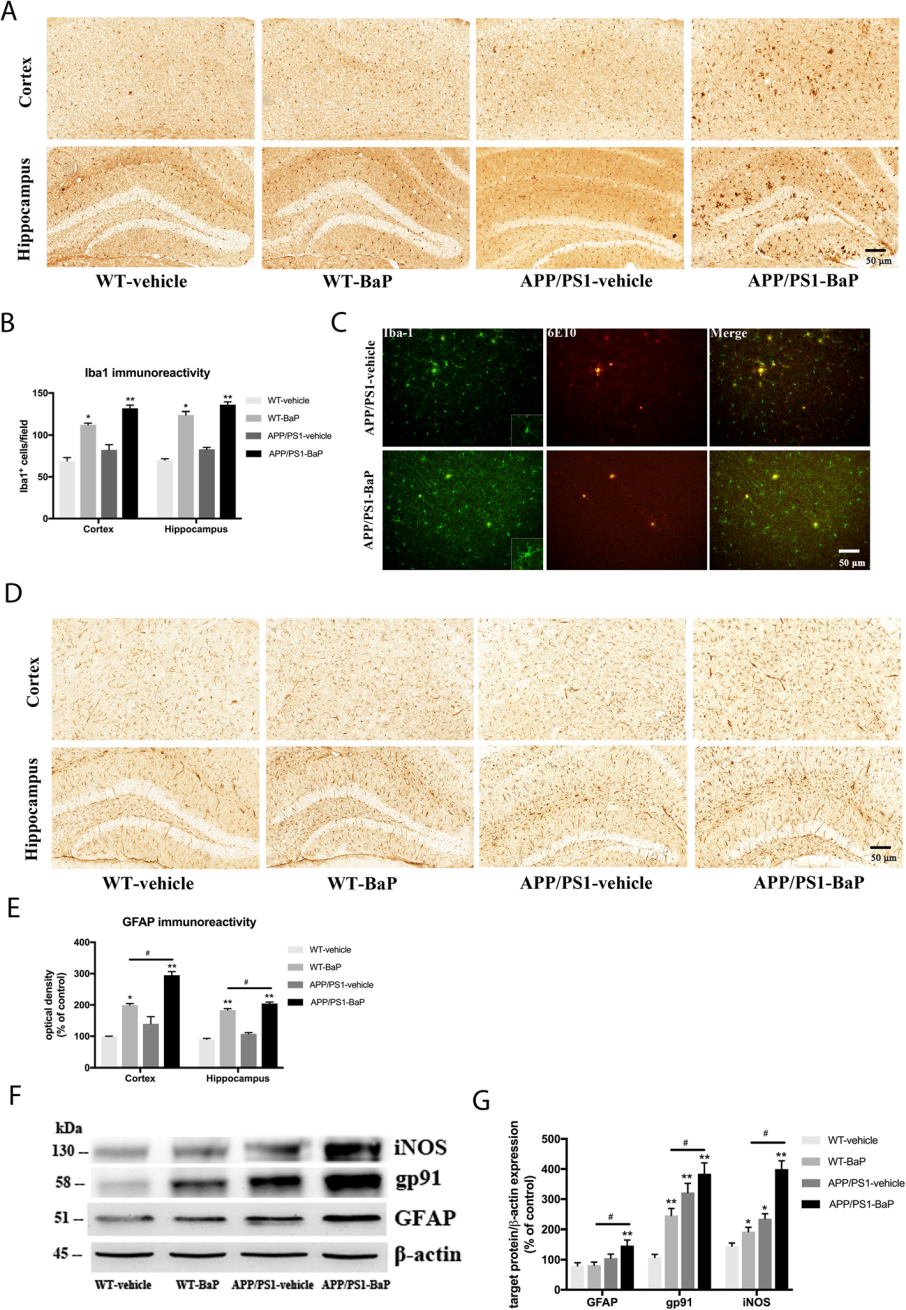
Glial activation and neuroinflammation were induced after BaP exposure. **a**, **b** Microglial activation was indicated by immunostaining with Iba-1 antibody (**a**) and counting of Iba-1-positive cells (**b**) in the cortex and the hippocampus. **c** Confocal double-staining of microglia and Aβ plaques by anti-Iba1 and anti-Aβ antibodies in the hippocampus. **d** Astroglial activation was detected with anti-GFAP antibody. **e** The GFAP immunoreactivity was measured and expressed as a percentage of the WT-Vehicle. **f**, **g** Levels of iNOS, gp91, and GFAP (three widely used neuroinflammatory markers) were detected by Western blot. β-Actin was used to monitor loading errors. Data are expressed as percentage of WT-Vehicle and are mean ± SEM of 3–4 mice in each treatment group. Significance was determined by two-way ANOVA followed by LSD multiple comparisons post-hoc test. *P < 0.*05*, **P < 0.*01* compared with WT-Vehicle control, ^#^*P < 0*.*05* compared with WT-BaP group

As shown in Fig. 6a and b, in comparison to vehicle controls, the number of Iba1^+^ microglia in the cortex was increased by 62.7% and 90.8% in WT-BaP mice and APP/PS1-BaP mice respectively, and microglial number in the hippocampus increased by 77.1% and 94.3% in WT-BaP mice and APP/PS1-BaP mice. Confocal double-label immunofluorescence also showed more prominent microglial activation in APP/PS1 mice after BaP exposure (Fig. 6c). Activated microglia in BaP-exposed mice appeared to have larger cell body and stronger staining of Iba1 (Fig. 6a, c). In addition, astroglia displayed remarkable increase in the immunoreactivity for GFAP (an astroglial marker) in both WT and APP/PS1 mice after BaP exposure, and APP/PS1-BaP mice exhibited higher immunoreactivity for GFAP than WT-BaP mice (Fig. 6d, e). These results indicated that BaP induced severer astrogliosis in APP/PS1 mice. Collectively, BaP exposure induced activation of microglia and astroglia in the cortex and the hippocampus. Meanwhile, Western blot analysis revealed significant upregulation of gp91, iNOS, and GFAP (three widely used neuroinflammatory markers) in BaP-treated mice (Fig. 6f, g). These results together indicated that chronic BaP exposure led to chronic neuroinflammation.

### BaP promoted neurodegeneration triggered by aged Aβ

**Fig. 7:**
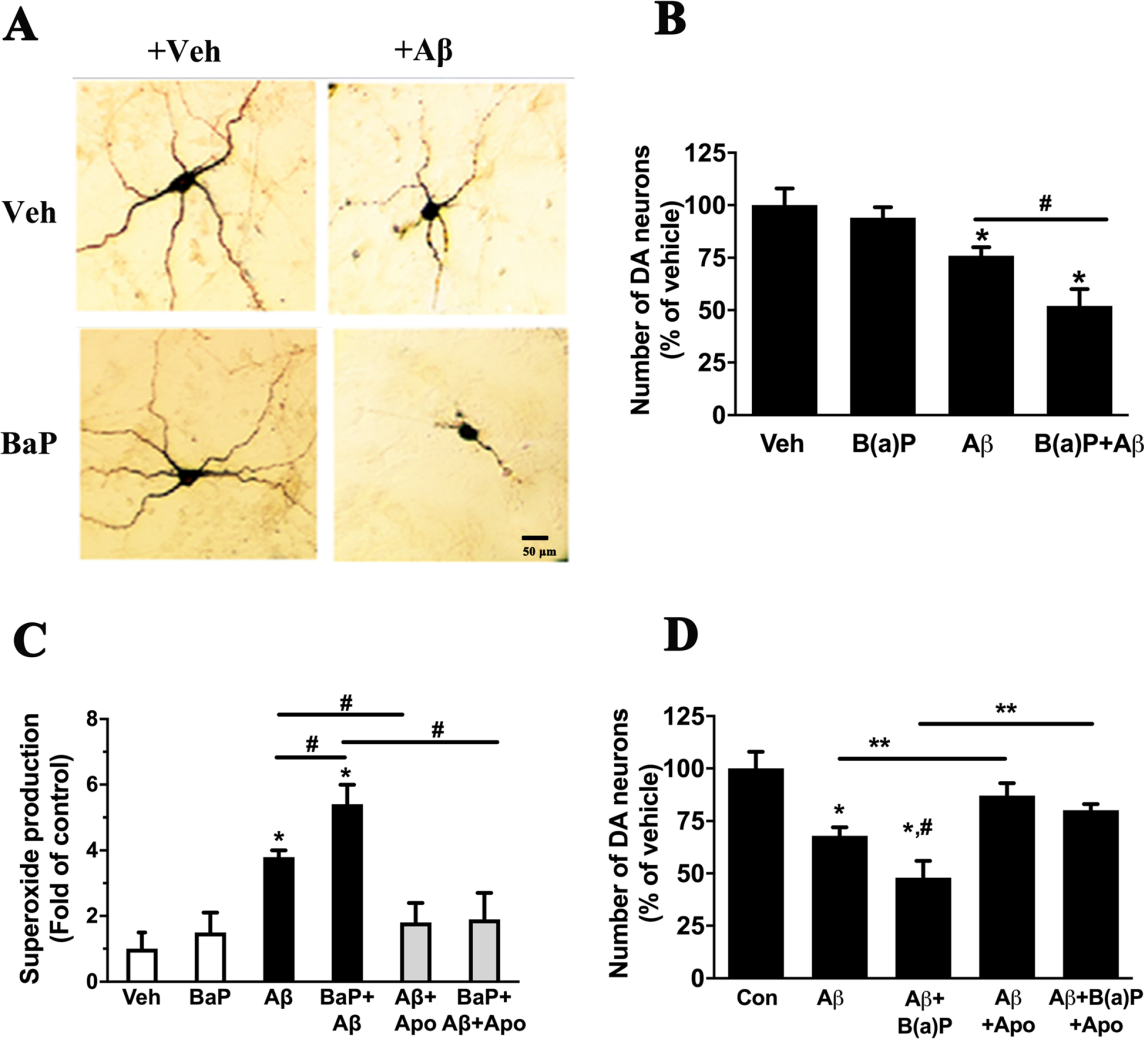
Inhibition of NADPH oxidase prevented neuronal loss induced by aged Aβ alone or in combination with BaP. **a**, **b** In primary neuron-glia cultures, loss of TH-positive neurons was examined by immunostaining (**a**) and cell counting (**b**) at 7 days after treatment with 5 μg/ml (1.108 μM) aged Aβ (a mixture of monomers, oligomers, and fibrils) and/or BaP (0.1 μM). **c** Extracellular superoxide production in primary neuron-glia cultures treated with Aβ and/or BaP with or without pre-treatment for 30 min with NADPH oxidase inhibitor apocynin (Apo, 0.25 mM) was measure at 24 h after Aβ/BaP treatment by SOD-inhibitable reduction of WST-1. **d** Neuroprotective effects of pre-treatment with Apo (0.25 mM) for 30 min against neurotoxic effects of Aβ and BaP determined by immunostaining and cell counting at 7 days after Aβ/BaP treatment. Data are expressed as fold or percent of vehicle-treated control and are mean ± SEM of 3–4 experiments performed in quadruplicate. Significance was determined by one-way ANOVA (**b**) or two-way ANOVA (**c** and **d**) followed by LSD multiple comparisons post-hoc test. *P < 0.*05* compared with vehicle-treated control, ^#^*P < 0*.*05* compared with Aβ-treated cultures. **P < 0.*05*

We then investigated whether neuroinflammation, especially the activation of NADPH oxidase, was important for BaP/Aβ-induced neurodegeneration. After 7-day treatment, aged Aβ peptide (5 μg/ml; 1.108 μM), which was prepared to form a mixture of monomer, oligomers and fibrils by incubation in PBS at 37 °C for 24 h, caused severe neurite injuries and shrunken bodies of TH-positive neurons in primary neuron-glia cultures. Such neuronal damages became much worse after the cultures were co-treated with Aβ and 0.1 μM BaP (Fig. 7a). Treatment with aged Aβ for 7 days led to 25% loss of TH-positive neurons, while BaP promoted such neuronal loss to 49% (Fig. 7b). Thus, BaP promoted neurodegeneration induced by aged Aβ.

NADPH oxidase has been shown to play a pivotal role in Aβ-induced neurotoxicity [28–31]. Our array results showed significant upregulation of multiple subunits of NADPH oxidase in the cortex of BaP-exposed mice (Table 1). Western blot results also revealed upregulation of gp91 (the catalytic subunit of NADPH oxidase) in the cortex/hippocampus of BaP-exposed mice. Therefore, we investigated whether upregulated NADPH oxidase mediated Aβ/BaP-induced neurodegeneration. We first measured extracellular superoxide production in neuron-glia cultures treated with Aβ and/or BaP. BaP and aged Aβ showed synergistic effects on extracellular superoxide production (Fig. 7c). Moreover, pre-treatment with NADPH oxidase inhibitor apocynin (0.25 mM) for 30 min before treatment with aged Aβ alone or in combination with BaP only blocked extracellular superoxide production but also neuronal loss, which were detected at 1 day or 7 days after Aβ/BaP treatment respectively (Fig. 7c, d). Neuroprotective effects of NADPH oxidase inhibitor on neurodegeneration induced by BaP and aged Aβ indicated that the upregulation and the activation of NADPH oxidase and resultant oxidative stress were important mediators of BaP/Aβ-induced neuronal loss seen in both in vitro and in vivo studies.

## Discussion

As a common and potent environmental organic pollutant, benzo(a)pyrene (BaP) was widely investigated for its adverse health effects. Besides carcinogenicity, neurotoxicity was also emerging as the crucial adverse effects of BaP exposure. Previous studies have demonstrated that BaP exposure during early life stage affects neurodevelopmental process, such as neuritis formation or neurodifferentiation [32, 33]. However, effects of BaP on adult brains warrant further investigation. Studies have shown that BaP exposure is associated with learning and memory deficits or some AD-like pathological changes such as Tau hyperphosphorylation. Therefore, it is essential to understand whether BaP, as an environmental risk factor of AD, can affect the disease progression of AD, especially neurodegeneration in the cortex and the hippocampus, the fundamental feature of AD. In this study, we evaluated the potential role of BaP exposure from the initial phase of AD using APP/PS1 and WT mice. We found that BaP exposure remarkably accelerated progressive cognitive and learning decline in both genotypes. BaP exposure exacerbated Aβ burden and plaque formation as well as neuroinflammation in APP/PS1 mice. Aβ/BaP-induced neurodegeneration could be mainly mediated through elevated neuroinflammation and NADPH oxidase activation.

In this study, APP/PS1 transgenic mice were used to evaluate effects of BaP on AD onset and progression. APP/PS1 mice expressing a chimeric mouse/human amyloid precursor protein (Mo/HuAPP695swe) and a mutant human presenilin 1 (PS1-dE9) were often used as an early-onset AD model. The time of onset of cognitive decline in APP/PS1 mice varies. Indeed, many studies have performed behavior tests and have shown cognitive decline in APP/PS1 mice when the mice were 8–12 month or even older [34, 35]. However, it has been reported that cognitive deficits in the Morris water maze test in the same APP/PS1 transgenic mice overexpressing human APP^K595N,M596L^/PSEN1^ΔE9^ (also called APP^K670N, M671L^/PSEN1^ΔE9^) emerge as early as 4 to 6 months and worsen with age [36–42]. BaP was capable to induce the neurobehavioral dysfunction in both APP/PS1 mice and WT mice, while such decline in APP/PS1 mice was earlier and severer than that in WT mice (Figs. 2 and 3). The neuronal loss and Aβ deposits in the hippocampus and the cortex were also observed in APP/PS1 mice after 2-month BaP exposure. BaP-induced neuronal loss in 7-month APP/PS1 mice (Fig. 4) promoted cognitive decline seen in APP/PS1 mice (Figs. 2 and 3). This two-hit model of AD with both genetic predisposition (namely mutant APP/PS1 genes) and exposure to environmental pollutant BaP recapitulated key features of AD including neuronal loss. It might imply that BaP can fasten the pathogenesis process and disease progression in people who carry AD susceptible genes.

The behavior changes without Aβ aggregation or neuronal loss/death in WT mice also indicated that BaP could initiate early neuronal damages and functional defects, such as neurotransmitter disturbances as well as synapse loss and dysfunction. Indeed, gene expression profiles showed significant downregulation of agrin and Slc6a4 (SERT) and remarkable upregulation of Ache in BaP-exposed mice (Table 1). All three proteins have been implicated in synaptic formation in AD patients or models [21–23]. Importantly, multiple studies have shown that early synaptic plasticity deficits or synapse loss, the disruption of neurotransmitters (e.g., serotonin and dopamine), and Aβ deposition in APP/PS1 mice correlate with cognitive decline [43–47]. In particular, synaptic dysfunction has been believed to be one of the major contributors to AD pathogenesis. Synapse loss in the hippocampus in the APP/PS1 mice occurs by 4 months of age [44]. Cognitive decline at age of 5 and 7 months in WT-BaP mice and APP/PS1-Vehicle (Fig. 2) might be partially due to synaptic defects and/or neurotransmitter imbalance but not loss/death of neurons.

Accumulating evidence has highlighted that neuroinflammation acts as an early pathophysiologic factor in the chronic progressive Aβ metabolism dysfunction and neuron loss in AD [48, 49]. Our results from the gene expression profile revealed a valuable picture of inflammation and immunoregulatory process, Aβ secretases, and degradation disturbance in the cortex after BaP exposure. Our results also revealed microglial activation and upregulation of pro-inflammatory cytokines (such as TNFα, IL1β, and IL6) after BaP exposure. Previous studies showed that pro-inflammatory cytokines, such as TNFα, IL6, and IL1β, could potentially disturb the Aβ clearance and cause memory impairments [48–50]. Besides, chemokine CCL2 was also associated with Aβ deposit in AD mice accompanied with aggravated behavior ability decline [51]. Previous studies also indicated that iNOS in AD mice was associated with plaque deposition and inflammation. The elevated NO production by upregulating iNOS in microglia could cause neurotoxicity, while the deficiency of iNOS showed protective effects in AD mice [52]. Consistent with these findings, in this study, both iNOS mRNA and protein were significantly elevated in both WT and APP/PS1 mice after BaP exposure. It suggested that iNOS could be an important inflammatory mediator in BaP neurotoxicity. Taken together, neuroinflammation could be initiated by BaP exposure and then induces the neurodegenerative process.

Our in vivo results showed that 2-month BaP exposure led to activation of microglia and astrocytes (Fig. 6 and Table 1). Lactational exposure to BaP induces astroglial activation and anxiety-like behavior in mice [53]. Subchronic oral administration of BaP (2 mg/kg/day for 28 days) causes astroglial reaction and motor/cognitive impairments in rats [54]. A previous study has shown that treatment of BV2 (mouse microglial cell line) with BaP (10 μM) triggered inflammatory responses, such as upregulation of iNOS and cyclooxygenase-2 (COX-2) as well as production of multiple pro-inflammatory factors including nitric oxide (NO), reactive oxygen species (ROS), IL-1β, and IL-6 [55]. Benzo[a]pyrene diol epoxide, the major metabolite of BaP, has been shown to upregulate COX-2 expression in rat astroglia [56]. Multiple lines of evidence implicated participation of upregulation and activation of NADPH oxidase in BaP-induced or BaP-amplified neuronal damages (Table 1, Fig. 6f and Fig. 7c, d). NADPH oxidase is mainly expressed in microglia and is the major source of extracellular superoxide during inflammation [29, 31, 57]. These findings together implied that microglial activation may be important for effects of BaP observed in this study with involvement of astroglia and/or neurons.

Previous studies including ours have shown that NADPH oxidase plays a pivotal role in Aβ-induced neurotoxicity [29–31, 58]. NADPH oxidase is mainly expressed in microglia [31]. Upregulated and/or over-activated NADPH oxidase is a major source of oxidative stress and oxidative neuronal damages during neuroinflammation and has been implicated as a therapeutic target for neurodegenerative diseases including AD [58]. In neuron-glia cultures, while aged Aβ (a mixture of monomers, oligomers, and fibrils) induced neuronal death, Aβ and “non-toxic” dose of BaP together caused synergistic neurodegeneration (Fig. 7). Mechanistically, BaP and aged Aβ synergistically induced extracellular superoxide production (Fig. 7c). While gene expression profiling showed significant increases in subunits of NADPH oxidase including gp91, p47, p67, and p40 (Table 1), Western blot results also revealed upregulation of gp91 protein (the catalytic subunit of NADPH oxidase) in the cortex of WT mice with BaP exposure and APP/PS1 mice with or without BaP exposure (Fig. 6f). More importantly, pre-treatment with NADPH oxidase inhibitor apocynin (0.25 mM) for 30 min protected cultured neurons against neurotoxicity of Aβ alone or in combination with BaP (Fig. 7d). Therefore, activation of NADPH oxidase and resultant oxidative stress were critical mechanisms for neuronal death induced by Aβ alone or combined with BaP. NADPH oxidase could be a crucial mediator for BaP-induced pathogenic process of AD.

## Conclusions

In summary, this study showed that BaP exposure induced and accelerated the progression of AD, in which neuroinflammation could be an early key event. Findings from this study suggested that BaP was an environmental risk factor for AD neurodegenerative diseases.

## Supplementary information

**Additional file 1: **Supplemental **Table S1.** Mouse Alzheimer’s Disease RT2 Profiler PCR Array.

## Data Availability

The datasets used and/or analyzed during the current study are available from the corresponding author on reasonable request.

## Abbreviations

BaP: Benzo(a)pyrene
AD: Alzheimer’s disease
APP/PS1: APPswe/PS1dE9
WT: Wildtype
Aβ: Beta-amyloid
PAHs: Polycyclic aromatic hydrocarbons
Iba1: Ionized calcium-binding adapter molecule 1
GFAP: Glial fibrillary acidic protein
NADPH: Nicotinamide adenine dinucleotide phosphate oxidase
BDNF: Brain-derived neurotrophic factor
GDNF: Glial cell line-derived neurotrophic factor
