# Supplementary material for: Benzo(a)pyrene exposure induced neuronal loss, plaque deposition, and cognitive decline in APP/PS1 mice
Source: J Neuroinflammation. 2020 Aug 31;17:258. doi: 10.1186/s12974-020-01925-y (PMC7461337; doi:10.1186/s12974-020-01925-y)
Supplement: Supplementary file 1 — Additional file 1: Supplemental Table S1. Mouse Alzheimer’s Disease RT2 Profiler PCR Array. [file 12974_2020_1925_MOESM1_ESM.docx]

**Supplemental table 1 Mouse Alzheimer’s Disease RT^2^ Profiler PCR Array**

| NCBI Ref Seq | Gene Symbol | Gene Name | Gene Function Category |
| --- | --- | --- | --- |
| NM_009598.1 | Ace | angiotensin I converting enzyme (peptidyl-dipeptidase A) 1 | Beta-Amyloid Generation, Oligomerization, Clearance, and Degradation |
| NM_009599.3 | Ache | acetylcholinesterase |  |
| NM_177583.4 | Aph1b | anterior pharynx defective 1b homolog (C. elegans) |  |
| NM_009696.3 | Apoe | apolipoprotein E |  |
| NM_007471.2 | App | amyloid beta (A4) precursor protein |  |
| NM_011792.4 | Bace1 | beta-site APP cleaving enzyme 1 |  |
| NM_009738.3 | Bche | butyrylcholinesterase |  |
| NM_007798.2 | Ctsb | cathepsin B |  |
| NM_009983.2 | Ctsd | cathepsin D |  |
| NM_031156.2 | Ide | insulin degrading enzyme |  |
| NM_008604 | Nep | Neprilysin |  |
| NM_008512.2 | Lrp1 | low density lipoprotein receptor-related protein 1 |  |
| NM_008514.3 | Lrp6 | low density lipoprotein receptor-related protein 6 |  |
| NM_001080926.1 | Lrp8 | low density lipoprotein receptor-related protein 8, apolipoprotein e receptor |  |
| NM_008604.3 | Mme | membrane metallo endopeptidase |  |
| NM_008604.3 | Ncstn | nicastrin |  |
| NM_008872.1 | Plat | plasminogen activator, tissue |  |
| NM_008873.2 | Plau | plasminogen activator, urokinase |  |
| NM_008943.2 | Psen1 | presenilin 1 |  |
| NM_011183.1 | Psen2 | presenilin 2 |  |
| NM_025498.2 | Psenen | presenilin enhancer 2 homolog (C. elegans) |  |
| NM_011436.3 | Sorl1 | sortilin-related receptor, LDLR class A repeats-containing |  |
| NM_009696.3 | Apoe | apolipoprotein E | Microtubule and Cytoskeleton Reorganization |
| NM_001038609.1 | Mapt | microtubule-associated protein tau |  |
| NM_009599.3 | Ache | acetylcholinesterase | Synaptic Formation |
| NM_021604.2 | Agrn | agrin |  |
| NM_009696.3 | Apoe | apolipoprotein E |  |
| NM_001048139.1 | Bdnf | brain derived neurotrophic factor |  |
| NM_010275.2 | Gdnf | glial cell line derived neurotrophic factor |  |
| NM_033217.3 | Ngfr | nerve growth factor receptor (TNFR superfamily, member 16) |  |
| NM_148938.2 | Slc1a3 | solute carrier family 1 (glial high affinity glutamate transporter), member 3 |  |
| NM_011773.2 | Slc30a3 | solute carrier family 30 (zinc transporter), member 3 |  |
| NM_010484.1 | Slc6a4 | solute carrier family 6 (neurotransmitter transporter, serotonin), member 4 |  |
| NM_001042451.1 | Snca | synuclein, alpha |  |
| NM_013454.3 | Abca1 | ATP-binding cassette, sub-family A (ABC1), member 1 | Cholesterol Metabolism |
| NM_009696.3 | Apoe | apolipoprotein E |  |
| NM_053272.2 | Dhcr24 | 24-dehydrocholesterol reductase |  |
| NM_001080926.1 | Lrp8 | low density lipoprotein receptor-related protein 8, apolipoprotein e receptor |  |
| NM_009696.3 | Apoe | apolipoprotein E | Lipid and Lipoprotein Metabolism |
| NM_013492.1 | Clu | clusterin |  |
| NM_016763.2 | Hsd17b10 | hydroxysteroid (17-beta) dehydrogenase 10 |  |
| NM_008512.2 | Lrp1 | low density lipoprotein receptor-related protein 1 |  |
| NM_001080926.1 | Lrp8 | low density lipoprotein receptor-related protein 8, apolipoprotein e receptor |  |
| NM_008875.3 | Pld1 | phospholipase D1 |  |
| NM_011198.3 | Ptgs2 | prostaglandin-endoperoxide synthase 2 |  |
| NM_001042451.1 | Snca | synuclein, alpha |  |
| NM_205769.1 | Crh | corticotropin releasing hormone | Hormone and Hormone Processing |
| NM_008171.3 | Grin2b | glutamate receptor, ionotropic, NMDA2B (epsilon 2) |  |
| NM_172812.2 | Htr2a | 5-hydroxytryptamine (serotonin) receptor 2A |  |
| NM_009696.3 | Apoe | apolipoprotein E | Apoptosis |
| NM_007471.2 | App | amyloid beta (A4) precursor protein |  |
| NM_007537.1 | Bcl2l2 | BCL2-like 2 |  |
| NM_009810.2 | Casp3 | caspase 3 |  |
| NM_007609.2 | Casp4 | caspase 4, apoptosis-related cysteine peptidase |  |
| NM_009811.3 | Casp6 | caspase 6 |  |
| NM_013492.1 | Clu | clusterin |  |
| NM_007987.1 | Fas | Fas (TNF receptor superfamily member 6) |  |
| NM_010554.4 | Il1a | interleukin 1 alpha |  |
| NM_001038609.1 | Mapt | microtubule-associated protein tau |  |
| NM_144931.2 | Nae1 | NEDD8 activating enzyme E1 subunit 1 |  |
| NM_054056.2 | Pawr | PRKC, apoptosis, WT1, regulator |  |
| NM_008943.2 | Psen1 | presenilin 1 |  |
| NM_011183.1 | Psen2 | presenilin 2 |  |
| NM_025498.2 | Psenen | presenilin enhancer 2 homolog (C. elegans) |  |
| NM_013693.2 | Tnf | tumor necrosis factor |  |
| NM_007668.3 | Cdk5 | cyclin-dependent kinase 5 | Cell Cycle Regulators |
| NM_007778.4 | Csf1 | colony stimulating factor 1 (macrophage) |  |
| NM_009971.1 | Csf3 | colony stimulating factor 3 (granulocyte) |  |
| NM_010113.2 | Egf | epidermal growth factor |  |
| NM_010248.1 | Gab2 | growth factor receptor bound protein 2-associated protein 2 |  |
| NM_008083.2 | Gap43 | growth associated protein 43 |  |
| NM_008175.3 | Grn | granulin |  |
| NM_010554.4 | Il1a | interleukin 1 alpha |  |
| NM_144931.2 | Nae1 | NEDD8 activating enzyme E1 subunit 1 |  |
| NM_011057.3 | Pdgfb | platelet derived growth factor, B polypeptide |  |
| NM_011577.1 | Tgfb1 | transforming growth factor, beta 1 |  |
| NM_007668.3 | Cdk5 | cyclin-dependent kinase 5 | Protein Kinases |
| NM_019827.6 | Gsk3b | glycogen synthase kinase 3 beta |  |
| NM_020009.2 | Mtor | mechanistic target of rapamycin (serine/threonine kinase) |  |
| NM_203491.1 | Chrm2 | cholinergic receptor, muscarinic 2, cardiac | Cell Signaling Molecules |
| NM_007390.3 | Chrna7 | cholinergic receptor, nicotinic, alpha polypeptide 7 |  |
| NM_023913.2 | Ern1 | endoplasmic reticulum (ER) to nucleus signalling 1 |  |
| NM_031156.2 | Ide | insulin degrading enzyme |  |
| NM_010554.4 | Il1a | interleukin 1 alpha |  |
| NM_008514.3 | Lrp6 | low density lipoprotein receptor-related protein 6 |  |
| NM_144931.2 | Nae1 | NEDD8 activating enzyme E1 subunit 1 |  |
| NM_008604.3 | Ncstn | nicastrin |  |
| NM_008873.2 | Plau | plasminogen activator, urokinase |  |
| NM_008875.3 | Pld1 | phospholipase D1 |  |
| NM_008943.2 | Psen1 | presenilin 1 |  |
| NM_011183.1 | Psen2 | presenilin 2 |  |
| NM_025498.2 | Psenen | presenilin enhancer 2 homolog (C. elegans) |  |
| NM_001081549.1 | Rcan1 | regulator of calcineurin 1 |  |
| NM_011163.3 | Eif2ak2 | eukaryotic translation initiation factor 2-alpha kinase 2 | Transcription regulators |
| NM_013672.2 | Sp1 | trans-acting transcription factor 1 |  |
| NM_007471.2 | App | amyloid beta (A4) precursor protein | Other Genes Involved in Alzheimer's disease |
| NM_009976.3 | Cst3 | cystatin C |  |
| NM_010010.1 | Cyp46a1 | cytochrome P450, family 46, subfamily a, polypeptide 1 |  |
| NM_016763.2 | Hsd17b10 | hydroxysteroid (17-beta) dehydrogenase 10 |  |
| NM_010927.3 | Nos2 | nitric oxide synthase 2, inducible |  |
| NM_011076.2 | Abcb1a | ATP-binding cassette, sub-family B (MDR/TAP), member 1A | blood-brain barrier permeability |
| NM_007399.3 | Adam10 | a disintegrin and metallopeptidase domain 10 | biological processes including cell signaling, adhesion and migration |
| NM_009615.5 | Adam17 | a disintegrin and metallopeptidase domain 17 |  |
| NM_010217.1 | Ctgf | connective tissue growth factor |  |
| NM_010493.2 | Icam1 | intercellular adhesion molecule 1 |  |
| NM_178678.4 | Lrrtm3 | leucine rich repeat transmembrane neuronal 3 |  |
| NM_023371.3 | Pin1 | protein (peptidyl-prolyl cis/trans isomerase) NIMA-interacting 1 |  |
| NM_011261.2 | Reln | reelin |  |
| NM_007426.3 | Angpt2 | angiopoietin 2 | Angiogenesis |
| NM_008584.3 | Meox2 | mesenchyme homeobox 2 |  |
| NM_007482.3 | Arg1 | arginase,liver | Immunoregulatory and inflammatory processes |
| NM_013653.3 | Ccl5 | chemokine (C-C motif) ligand 5 |  |
| NM_013654.2 | Ccl7 | chemokine (C-C motif) ligand 7 |  |
| NM_007643.3 | Cd36 | CD36 antigen |  |
| NM_011616.2 | Cd40lg | CD40 ligand |  |
| NM_007807.5 | Gp91 | cytochrome b-245, beta polypepetide |  |
| NM_010554.4 | Il1a | interleukin 1 alpha |  |
| NM_008361.4 | Il1β | interleukin 1 beta |  |
| NM_010556.4 | Il3 | interleukin 3 |  |
| NM_031168.1 | Il6 | interleukin 6 |  |
| NM_010957.3 | Ogg1 | 8-oxoguanine DNA-glycosylase 1 |  |
| NM_010877.5 | P67 | neutrophil cytosolic factor 2 |  |
| NM_001286037.1 | P47 | neutrophil cytosolic factor 1 |  |
| NM_008677.2 | P40 | neutrophil cytosolic factor 4 |  |
| NM_001313922.1 | iNOS | nitric oxide synthase 2, inducible |  |
| NM_023371.3 | Pin1 | protein (peptidyl-prolyl cis/trans isomerase) NIMA-interacting 1 |  |
| NM_011146.2 | Pparg | peroxisome proliferator activated receptor gamma |  |
| NM_011198.3 | Ptgs2 | prostaglandin-endoperoxide synthase 2 |  |
| NM_009438.5 | Rpl13a | ribosomal protein L13A |  |
| NM_013693.2 | Tnf | tumor necrosis factor |  |
| NM_001272078 | TREM2 | triggering receptor expressed on myeloid cells 2 |  |
| NM_028029.2 | Dnmbp | dynamin binding protein | Regulate the configuration of cell junctions |
| NM_011261.2 | Reln | reelin |  |
| NM_008054.1 | Fyn | Fyn proto-oncogene | proto-oncogene |
| NM_173048.2 | Gga3 | golgi associated, gamma adaptin ear containing, ARF binding protein 3 | unclassified |
| NM_010480.5 | Hsp90aa1 | heat shock protein 90, alpha (cytosolic), class A member 1 |  |
| NM_001025360.2 | Klc1 | kinesin light chain 1 |  |
| NM_145933.3 | St6gal1 | beta galactoside alpha 2,6 sialyltransferase 1 |  |
